# Neoadjuvant Tyrosine Kinase Downstaging of T2 Renal Cell Carcinoma in Solitary Kidney Before Robotic Partial Nephrectomy

**DOI:** 10.1089/cren.2015.0005

**Published:** 2015-11-01

**Authors:** Mary K. Powers, Oliver Sartor, Benjamin R. Lee

**Affiliations:** Department of Urology, Tulane University School of Medicine, New Orleans, Louisiana.

## Abstract

We highlight the use of a tyrosine kinase inhibitor, pazopanib, for neoadjuvant downstaging a 7.4 cm right biopsy-proven clear cell renal-cell carcinoma in a solitary kidney before surgical intervention of robotic partial nephrectomy with retrograde cooling to induce cold ischemia in a 79-year-old male.

## Clinical History

This case describes a 79-year-old male with history of recurrent renal-cell carcinoma with initial presentation in 2005. He underwent a left laparoscopic radical nephrectomy with intracorporeal morcellation at an outside hospital that demonstrated clear cell renal-cell carcinoma. He was lost to follow-up, and on subsequent surveillance imaging 9 years later, he was found to have a 7.4 cm right upper pole renal mass on CT scan. The tumor abutted the collecting system and extended caudally below the renal hilum, but no renal vein thrombus was noted.

He underwent two percutaneous biopsies, the first being negative for malignancy. His second biopsy confirmed clear cell renal-cell carcinoma. The patient denied any history of gross hematuria, flank pain, or weight loss. In addition, the patient has a history of coronary artery disease, stroke, and two myocardial infarctions, for which he has been maintained on Plavix.

## Physical Examination

On physical examination, his abdomen was remarkable for laparoscopic scars caused by his previous left laparoscopic radical nephrectomy as well as left inguinal incision, which the patient was unsure of the etiology of this scar. His right renal mass was not palpable despite his body mass index of 20.3. He did not have any signs of venous thrombus, including lower extremity edema or a pathologic varicocele. His preoperative creatinine was 1.2 mg/dL with glomerular filtration rate (GFR) of 69 and his remaining laboratories were unremarkable.

## Diagnosis

**FIG. 1.**
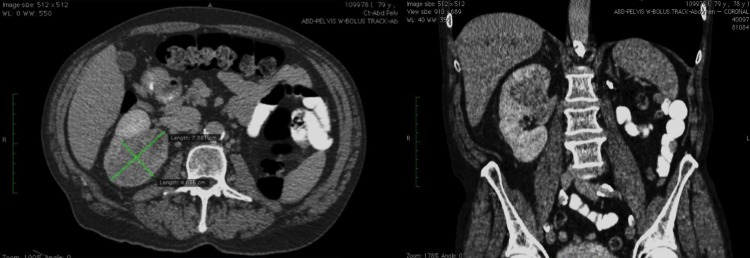
Pretreatment axial-contrasted CT scan of the abdomen and pelvis demonstrating right renal mass. Coronal view of 7.4 cm right upper pole renal-cell carcinoma extending caudally involving >50% of solitary kidney.

Based on the patient's history of clear cell renal-cell carcinoma in his contralateral kidney, contrasted CT imaging demonstrating a sizable 7.4 cm enhancing right renal mass was highly concerning a recurrent malignant process, likely renal-cell carcinoma (Fig. 1). Secondary to his solitary kidney, confirmatory biopsy was performed. Although his first biopsy was negative, his second renal biopsy demonstrated clear cell renal-cell carcinoma, Furhman grade 1, positive immunohistochemical stains for vimentin and CD10, and negative for epithelial membrane antigen.

## Intervention

The patient was counseled on his options that included active surveillance, but his risk of metastasis was considerable given the size of his tumor >4 cm, and radical nephrectomy that would subsequently commit him to end-stage renal failure and dialysis, of which the mortality rate in the first year would be significant. Additional options included robotic *vs* open partial nephrectomy, or to start a tyrosine kinase inhibitor (TKI) to help downstage the size of the mass and readdress for surgical intervention.

He was subsequently started on pazopanib for immunotherapy. This particular TKI was chosen for its reduced side effect profile compared with sunitinib or sorafenib, and for being better tolerated in patients. He was started on a regimen of 600 mg orally of pazopanib per day. After 3 months, he had a slight elevation in his liver function tests and the regimen was stopped for 2 weeks. Once his liver function tests normalized, he was restarted on a dose of 300 mg per day. The patient reported some loss of taste in his taste buds and sensed bitter taste in certain foods, but he remained relatively asymptomatic during his treatment course.

**FIG. 2.**
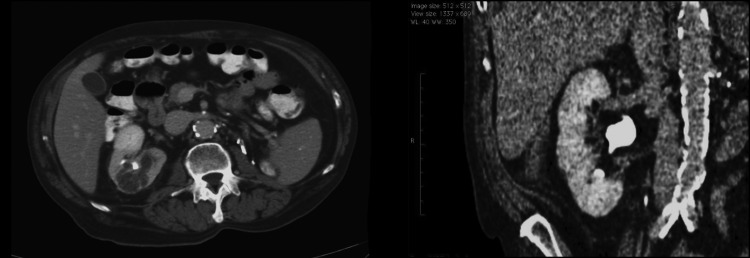
Post-treatment axial-contrasted abdominal CT scan demonstrating reduction in the right renal mass by 25%. Coronal view post-treatment of tyrosine kinase inhibitor. Regression of tumor away from the renal vein.

After 4 months of medical therapy, repeat axial imaging was obtained. There was a 25% reduction in his tumor size down to 3.7 × 5.5 cm and it had regressed away from the renal hilum (Fig. 2). The patient held his pazopanib therapy for 2 weeks before operation, as it can complicate wound healing. He underwent effective right robotic partial nephrectomy with retrograde renal cooling^1^ with a total renal clamp time of 28 minutes and estimated blood loss of 600 mL. At the termination of the case, a ureteral stent was placed. It was noted during renorrhaphy that the TKI produced a sponge-like edematous change in the characteristic of the tissue.

## Follow-Up

Postoperatively the patient received two units of pack red blood cells. His serum creatinine peaked at 2.4 in the immediate postprocedural period. He convalesced in the hospital and was discharged on postoperative day 3. Pathology report demonstrated clear cell renal-cell carcinoma, 3.8 cm, Furhman grade 3, negative margins but renal sinus vein involvement, Stage T_3a_N_0_M_0_. Interestingly, there was a 50% reduction in size of the tumor on final pathology analysis.

Five days after operation, he returned to the emergency room with gross hematuria and clot retention. He underwent bedside irrigation and was placed on continuous bladder irrigation. Before angiography, Mucomyst was administered, IV hydration performed, and CO_2_ used for renal angiography to minimize contrast load by interventional radiology, which demonstrated a right lower pole pseudoaneurysm, which was effectively coiled. His serum creatinine peaked at 3.8 after coil embolization. His hematuria resolved and he was discharged.

Now the patient is 1 year after operation and he remains off of hemodialysis with a latest creatinine of 2.8. He followed for both nephrology for chronic disease and urology for surveillance for his history of renal-cell carcinoma, and currently has no evidence of recurrence.

## Outcomes

Neoadjuvant TKI therapy before surgical intervention has been described, particularly for use in tumor downstaging; however, there have been no reports of application in a solitary kidney before robotic partial nephrectomy. Studies demonstrate that sunitinib therapy can reduce renal cell tumor sizes from 21% to 55%.^2,3^ Delayed wound healing can result after TKI therapy. Previous studies have reported that urinary leaks occurred in 3 of 14 renal units, all of which were managed conservatively.^2^ The likely mechanism behind an increased rate of urine leak is inhibited angiogenesis factors, which are required for wound healing being inhibited by the TKI medication. Similarly, our patient required extensive surgical dissection to remove the tumor in its entirety and likely developed a pseudoaneurysm because of poor healing properties despite stopping pazopanib 2 weeks before his partial nephrectomy.

Silberstein and colleagues^2^ reported on the nonmetastatic disease patients; seven of seven were disease free at a mean follow-up of 24 months. Similarly, another study reported a phase II study of pazopanib in 25 patients, 96% of them underwent open surgery. Enrollment criteria included whether radical nephrectomy or partial nephrectomy was likely to yield GFR <30 mL/minute/1.73 m.^4^ Treatment before operation was a median of 10.6 weeks, with five of six patients having partial nephrectomies resulting in development of fistulas and one patient progressing to end-stage renal failure requiring dialysis.^4^ The high rate of urinary leaks as demonstrated by multiple studies is thought to be secondary to delayed wound healing associated with inhibition of vascular endothelial growth factor (VEGF) receptors, which is the main mechanism of TKI.^2,4,5^ Wound strength, re-epithelialization, and revascularization inhibition of VEGF signaling can all lead to impaired wound healing.^5^ Floseal^™^ hemostatic matrix was used intraoperatively during renorrhaphy to prompt the coagulation cascade and facilitate fibrin formation. Other risk factors associated with TKI use include hypertension, proteinuria, gastrointestinal perforation, thrombosis, cardiac impairment, or hypothyroidism.^5^ Further studies demonstrated comparable results with only 1 of 10 patients experiencing disease recurrence and that the patient effectively underwent tumor thrombectomy at 8 months postoperatively. Our patient is experiencing similar results with no evidence of tumor recurrence at 1 year. The use of tyrosine kinase therapy for neoadjuvant surgical downstaging of high-risk renal-cell carcinoma before robotic partial nephrectomy presents a viable option in these challenging cases.

## Abbreviations Used

CT: computed tomography
GFR: glomerular filtration rate
TKI: tyrosine kinase inhibitor
VEGF: vascular endothelial growth factor

## References

[1] Saitz TR, Dorsey PJ Jr., Colli J, Lee BR Induction of cold ischemia in patients with solitary kidney using retrograde intrarenal cooling: 2 year functional outcomes. Int Urol Nephrol 2013;45:313–320 23386246 10.1007/s11255-013-0391-5

[2] Silberstein J, Millard F, Mehrazin R, et al. Feasibility and efficacy of neoadjuvant sunitinib before nephron-sparing surgery. BJU Int 2010;106:1270–1276 20394613 10.1111/j.1464-410X.2010.09357.x

[3] Gorin M, Ekwenna O, Soloway M, et al. Dramatic reduction in tumor burden with neoadjuvant sunitnib prior to bilateral nephron-sparing surgery. Urology 2012;79:e11 21676439 10.1016/j.urology.2011.04.018

[4] Rini B, Uzzo RG, Cambell SC, et al. Phase II study of pazopanib to optimize preservation of renal parenchyma. J Urol 2015;194:297–303 25813447 10.1016/j.juro.2015.03.096

[5] Kamba T, McDonald DM Mechanisms of adverse effects of anti-VEGF therapy for cancer. Br J Cancer 2007;96:1788–1795 17519900 10.1038/sj.bjc.6603813 PMC2359962

